# Breast Cancer Drug Approvals Issued by EMA: A Review of Clinical Trials

**DOI:** 10.3390/cancers13205198

**Published:** 2021-10-16

**Authors:** Simona Duranti, Alessandra Fabi, Marco Filetti, Rosa Falcone, Pasquale Lombardi, Gennaro Daniele, Gianluca Franceschini, Luisa Carbognin, Antonella Palazzo, Giorgia Garganese, Ida Paris, Giovanni Scambia, Antonella Pietragalla

**Affiliations:** 1 Scientific Directorate, Fondazione Policlinico Universitario A. Gemelli IRCCS, 00168 Rome, Italy; alessandra.fabi@policlinicogemelli.it (A.F.); gennaro.daniele@policlinicogemelli.it (G.D.); giovanni.scambia@policlinicogemelli.it (G.S.); antonella.pietragalla@policlinicogemelli.it (A.P.); 2 Unit of Precision Medicine in Breast Cancer, Scientific Directorate, Department of Woman and Child Health and Public Health, Fondazione Policlinico Universitario A. Gemelli IRCCS, 00168 Rome, Italy; 3 Phase 1 Unit, Fondazione Policlinico Universitario A. Gemelli IRCCS, 00168 Rome, Italy; marco.filetti@guest.policlinicogemelli.it (M.F.); rosa.falcone@policlinicogemelli.it (R.F.); pasquale.lombardi@guest.policlinicogemelli.it (P.L.); 4 Multidisciplinary Breast Center, Department of Woman and Child Health and Public Health, Fondazione Policlinico Universitario A. Gemelli IRCCS, Università Cattolica del Sacro Cuore, 00168 Rome, Italy; gianluca.franceschini@policlinicogemelli.it; 5 Department of Woman and Child Health and Public Health, Fondazione Policlinico Universitario A. Gemelli IRCCS, 00168 Rome, Italy; luisa.carbognin@guest.policlinicogemelli.it (L.C.); ida.paris@policlinicogemelli.it (I.P.); 6 Comprehensive Cancer Center, UOC di Oncologia Medica, Fondazione Policlinico Universitario A. Gemelli IRCCS, 00168 Rome, Italy; antonella.palazzo@policlinicogemelli.it; 7 Gynecology and Breast Care Center, Mater Olbia Hospital, 07026 Olbia, Italy; giorgia.garganese@materolbia.com; 8 Department of Life Science and Public Health, Università Cattolica del Sacro Cuore, 00168 Rome, Italy

**Keywords:** breast cancer, drugs approval, EMA

## Abstract

**Simple Summary:**

Considering the high incidence and mortality of breast cancer, a lot of trials evaluating new drugs for the treatment of this disease are currently ongoing. However, few drugs show a statistically and clinically significant improvement in the outcomes leading to the Competent Authority approval. In this review we analyzed the European Medicines Agency breast cancer drug indications issued between January 2015 and June 2021 and we evaluated the clinical trials results leading to the approval and their update.

**Abstract:**

Breast cancer represents the first cause of cancer worldwide and the leading cause of cancer mortality for women. Therefore, new therapies are needed to improve the prognosis of women diagnosed with this disease. In this review, we summarize the new drug indications for the treatment of breast cancer approved by European Medicines Agency between January 2015 and June 2021. In particular, we analyzed the clinical trials results leading to approvals and their update (when available), according to setting (localized and locally advanced or metastatic) and clinical features (hormone receptor positive, HER2 positive, triple negative, BRCA 1/2 mutation). The aim of this paper is to describe the clinical benefit obtained with the new indications.

## 1. Breast Cancer Landscape and European Authorization Procedure for New Drugs

Breast cancer represents the most frequent cancer and the leading cause of cancer-related mortality in females [1]. In this scenario, with 685,000 deaths globally in 2020 [2], breast cancer is the most prevalent tumor, representing a public health issue and a research priority.

Conventionally, this disease includes three major subtypes: hormone receptor positive, which represents about 70% of cases; human epidermal growth factor receptor 2 (HER2) positive (15–20%); and triple negative (15%). More than 90% of cases are not metastatic at diagnosis and for stage I, the 5-year breast cancer-specific survival is about 99% for hormone receptor-positive breast cancer, ≥94% in HER2-positive disease, and ≥85% in triple-negative patients. Instead, for metastatic disease, median overall survival is 4–5 years for the hormone receptor-positive subgroup, 5 years for HER2-positive disease, where the prognosis is remarkably improved with the specific therapies, and 10–13 months for triple negative [3].

Recently, in the context of a more personalized medicine approach, other actionable targets predictive of response to new therapies as BReast CAncer gene (BRCA) 1/2, phosphatidylinositol-4,5-bisphosphate 3-kinase, catalytic subunit alpha (PIK3CA) and programmed death-ligand 1 (PD-L1) entered in clinical practice. Moreover, the breast cancer treatment landscape is rapidly evolving with an important effort both of clinical research (profit and academic) and of regulatory agencies to quickly provide access to new therapeutic options for patients worldwide.

In Europe, a centralized authorization exists for the approval of new drugs. The centralized procedure is coordinated by European Medicines Agency (EMA), which works in a network with the competent authorities of each Member State. Through its Scientific Committee for Medicinal Products for Human Use (Committee for Human Medicinal Products, CHMP), EMA evaluates the documentation submitted by the pharmaceutical company, verifies the benefit/risk ratio based on the efficacy and safety data, and expresses an opinion within a predefined period of time (maximum 210 days). The opinion expressed by CHMP is sent to the European Commission, which issues a final decision on the marketing authorization of the medicinal product with a binding nature for all Member States. Medicines authorized by EMA are then marketed in individual Member States [4]. In Italy, in particular, a negotiation phase between the Italian Medicines Agency (AIFA) and the pharmaceutical companies of the reimbursement and price of the medicines paid by the National Health Service is envisaged.

The aim of this review is to summarize and analyze the trial results leading to the approval of breast cancer drugs issued by EMA between January 2015 and June 2021. An update of the trial results, when available, is also reported.

## 2. Methods

We searched all oncologic drugs with new indication or extension of indication mentioned on the EMA website in the section of CHMP meeting highlights that took place monthly between January 2015 and June 2021. We created a database with all new oncologic drug indications, year of approval, mechanism of action of the drug, if it was a new drug or an extension of indication, disease, setting, features of clinical trial (phase, primary endpoint, control arm), and results. For this review, we selected the drugs for which a new indication or an extension of indication for breast cancer treatment were issued. The European Public Assessment Report (EPAR), the main study presented by the company, the first publication of the trial, and the last update were evaluated. We analyzed all the clinical trials leading to the approvals, according to setting (localized and locally advanced or metastatic) and biological features (hormone receptor positive, HER2 positive, triple negative, BRCA mutated) and their update, with the aim of evaluating the clinical benefit of the new drug/combination versus the comparison arm (standard of care or placebo). The initial screening of the produced results was carried out by two authors (SD and APi) independently based on the title and abstract of the papers. All the works considered eligible were subjected to a full-text review. A separate consensus of the remaining investigators resolved any disagreement in the data selection phase.

## 3. Analyses of Clinical Trials and Update

In the reporting period, EMA issued 21 new indications for breast cancer drugs, based on 20 different clinical trials. No new approvals were reported for the first six months of 2021 (Table 1).

### 3.1. Localized and Locally Advanced Disease

In this setting, EMA issued five new indications for four drugs, all of them in HER2-positive disease. Clinical trials leading to the approvals are presented in Table 2.

The first drug approved was pertuzumab, which received two extensions of indication in the reported period. The first regarded the neoadjuvant setting in combination with trastuzumab and chemotherapy for HER2-positive, locally advanced, inflammatory, or early stage breast cancer at high risk of recurrence. This approval was based on the phase II NeoSphere and TRYPHAENA trial results. The NeoSphere trial evaluated four different arms of treatment in the neoadjuvant setting: trastuzumab plus docetaxel (group A), pertuzumab and trastuzumab plus docetaxel (group B), pertuzumab and trastuzumab alone (group C), or pertuzumab plus docetaxel (group D). The primary endpoint was pathological complete response (pCR), which showed significant improvement in patients treated with pertuzumab and trastuzumab plus docetaxel (45.8%), compared with those given trastuzumab and docetaxel (29.0%), pertuzumab plus docetaxel (24.0%), and pertuzumab and trastuzumab (16.8%) [5]. The update of 5-year analysis showed that 5-year progression-free survival (PFS) rates were 81% for group A, 86% for group B, 73% for group C, and 73% for group D. Patients who achieved pCR had longer PFS compared with patients who did not (85% vs. 76%) [6]. In the TRYPHAENA trial, patients were randomized to receive six neoadjuvant cycles of trastuzumab and pertuzumab in association with anthracycline and taxanes-based chemotherapy in two different schedules (arm A and B) or in association with carboplatin (arm C). The primary endpoint was the tolerability, in particular cardiac safety. The trial showed 2.7% of symptomatic left ventricular systolic dysfunction (LVSD) in arm B and a decline in left ventricular ejection fraction of ≥10% points from baseline to <50% in 5.6%, 5.3%, and 3.9% in arm A, B, and C, respectively [7]. The update of the trial showed 3 year PFS rates of 89%, 89%, and 87%, respectively, in the three arms of the treatment. Two (2.8%), three (4.0%), and four (5.4%) patients, respectively, had any-grade LVSD and eight (11.1%), 12 (16.0%), and nine (11.8%) experienced left ventricular ejection fraction declines ≥10% from baseline to <50% during post treatment follow up [8].

The second indication of pertuzumab was for the adjuvant treatment of HER2-positive early breast cancer patients at high risk of recurrence in combination with trastuzumab and chemotherapy, based on the phase III APHINITY trial results. In this trial, more than 4800 patients were randomized to receive pertuzumab or placebo in association with chemotherapy and trastuzumab. Disease recurrence occurred in 171 (7.1%) and 210 (8.7%) patients, respectively (HR 0.81, *p* = 0.045), and the estimates of the 3-year rates of invasive disease-free survival (IDFS) were 94.1% and 93.2%, respectively. Cardiac toxicity was infrequent in both arms and diarrhea of grade ≥3 occurred more frequently in the pertuzumab arm than with placebo (9.8% vs. 3.7%) [17]. At 74 months median follow-up, the overall survival (OS) did not reach statistical significance (6-year OS 95% versus 94% with 125 versus 147 deaths for pertuzumab and placebo, respectively). IDFS showed an HR of 0.76 and 6-year IDFS was 91% and 88%, respectively [18].

The second drug approved was neratinib, which received the indication for the extended adjuvant treatment of early stage hormone receptor-positive and HER2-positive breast cancer who completed adjuvant trastuzumab-based therapy less than one year before, based on the ExteNET trial results. In the study, after 2 years of follow-up, 70 IDFS events occurred in patients in the neratinib group and 109 in the placebo group, with a 2-year IDFS rate of 93.9% vs. 91.6%, respectively (HR 0.67, *p* = 0.0091). The most common grade 3–4 adverse events in patients in the neratinib arm were gastrointestinal events (diarrhea 41% vs. 2%, vomiting 3% vs. <1%, and nausea 2% vs. <1%). Serious adverse events were reported in 103 (7%) and 85 (6%) patients, respectively [19]. After a median follow-up of 5.2 years, the 5-year IDFS was 90.2% with neratinib and 87.7% with placebo (116 vs. 163 events, respectively, HR 0.73, *p* = 0.0083), with an absolute benefit of 5.1% in hormone receptor-positive patients [20]. Moreover, in this subgroup, patients obtained an absolute 8-year OS benefit of 2.1% [21].

Additionally, T-DM1 received an extension of indication in the adjuvant setting, in particular for the adjuvant treatment of adult patients with HER2-positive early breast cancer who have residual invasive disease after neoadjuvant taxane-based and HER2-targeted therapy. In the pivotal KATHERINE trial, 1486 patients treated with neoadjuvant taxane-based therapy with trastuzumab and residual invasive disease at surgery were randomized to receive 14 cycles of adjuvant T-DM1 or trastuzumab. The primary endpoint was IDFS and invasive disease or death occurred in 91 (12.2%) and 165 (22.2%) patients of the T-DM1 and trastuzumab arms, respectively. Estimated percentages of invasive-free patients at three years were 88.3% for the experimental arm and 77.0% for trastuzumab, and in particular, T-DM1 reduced the risk of recurrence or death by 50% (HR 0.50; *p* < 0.001). No new signals of toxicity were observed with T-DM1. Adverse events grade ≥ 3 occurred in 25.7% vs. 15.4% in the experimental and control arm, respectively, with a total of 18% vs. 2.1% of discontinuation. The most common adverse events in the first arm were decreased platelet count (5.7%) and hypertension (2.0%) [36].

The last approval in this setting regarded trastuzumab/pertuzumab subcutaneous formulation, based on the non-inferiority phase III FeDeriCa trial results. In the trial, this formulation was compared to intravenous pertuzumab plus trastuzumab for the neoadjuvant/adjuvant treatment of HER2-positive early breast cancer in terms of pharmacokinetics, efficacy, and safety. The study met the primary endpoint and in particular, the geometric mean ratio of pertuzumab serum Ctrough subcutaneous to serum Ctrough intravenous was 1.22. Moreover, similar pCR rates between the two treatment arms were reported (59.7% vs. 59.5% for the experimental and standard arm, respectively) [39].

### 3.2. Metastatic Setting

In the analyzed period, EMA issued 16 new indications concerning metastatic disease, of which 10 in hormone receptor-positive/HER2 negative, three in HER2-positive, one in triple-negative, and two in breast cancer with BRCA mutation. The trials leading to the approvals are reported in Table 3.

#### 3.2.1. Hormone Receptor Positive/HER2 Negative

In hormone receptor-positive/HER2-negative metastatic breast cancer among 10 new indications, seven regarded the CDK4/6 inhibitors palbociclib, ribociclib, and abemaciclib; two the hormonal agent fulvestrant; and one the PIK3CA inhibitor alpelisib.

The first CDK4/6 inhibitor approved was palbociclib, which received two indications, for the treatment of hormone receptor-positive/HER2-negative breast cancer in combination with an aromatase inhibitor or in combination with fulvestrant in patients who received prior endocrine treatment. The first approval derived from the results of the PALOMA-2 trial, where the drug, in association with letrozole, showed an improvement in median PFS of 10 months (24.8 months vs. 14.5 months, HR 0.58, *p* < 0.001) compared with letrozole alone in patients who had not received prior treatment for metastatic disease. The most common grade 3–4 adverse events were hematologic events, in particular neutropenia (66.4% vs. 1.4%, with 1.8% and 0% of febrile neutropenia, respectively), leukopenia (24.8% vs. 0%), and anemia (5.4% vs. 1.8%) [9]. The update of the trial after a median follow-up of 38 months showed a median PFS of 27.6 months compared to 14.5 months with letrozole alone [10].

The second approval of palbociclib was based on the PALOMA-3 results, where the drug was tested in association with fulvestrant for the treatment of patients with progression or relapse during previous endocrine therapy. The primary endpoint was PFS. The trial showed an advantage of about 5 months for the combination versus fulvestrant alone (9.2 vs. 3.8 months, HR 0.42; 95% CI, 0.32 to 0.56; *p* < 0.001), with no new changes regarding the safety profile [11]. The final protocol-specified OS analysis, at 44.8 months of follow-up, showed an advantage in OS of almost 7 months (34.9 vs. 28.0 months, HR 0.81, *p* = 0.09) for the combination arm compared to fulvestrant alone and of 10 months considering patients with sensitivity to previous endocrine therapy (39.7 vs. 29.7 months, HR 0.72) [12]. The last update of the trial, at 73.3 months of follow-up, showed that the improvement in OS continues to be observed, with an HR of 0.81, in particular, the 5-year OS rate was 23.3% with palbociclib combination and 16.8% with fulvestrant plus placebo [13].

The second CDK4/6 inhibitor receiving approval was ribociclib, for first-line treatment of postmenopausal hormone receptor-positive breast cancer in association with letrozole, based on MONALEESA-2 trial results, with PFS as the primary endpoint of the trial. The PFS rate was 63.0% in the ribociclib group and 42.2% in the placebo group with a median duration of PFS not reached and 14.7 months, respectively (HR 0.56, *p* = 3.29 × 10^−6^ for superiority). The most common grade 3–4 adverse events were neutropenia (59.3% vs. 0.9% in the placebo group) and leukopenia (21.0% vs. 0.6%) [15]. The second interim analysis at 26.4 months of follow-up showed a median PFS of 25.3 months for ribociclib plus letrozole and 16.0 months for the placebo group (HR 0.57, *p* = 9.63 × 10^−8^); OS data remained immature [16].

Furthermore, ribociclib received approval in association with fulvestrant in first- or second-line hormone therapy based on the MONALEESA-3 trial. In this study, 726 hormone receptor-positive breast cancer patients were randomized 2:1 to receive ribociclib or placebo with fulvestrant. Median PFS, the primary endpoint, was significantly improved by the experimental arm of 7.7 months (20.5 vs. 12.8, HR 0.59, *p* < 0.001). Grade 3 or 4 adverse events reported were hematologic events, observed in more than 50% of patients in the experimental arm (the majority of grade 3), almost absent in the fulvestrant arm [26]. The recent update of the trial with a median follow-up of 56.3 months showed that median OS was 53.7 months for the ribociclib arm versus 41.5 months for the placebo arm (HR 0.73). In the first-line setting, median OS was not reached for the ribociclib arm with about 60% of patients alive at the median follow-up, while median OS was 51.8 months in the placebo arm (HR 0.64). In the second-line setting, median OS was 39.7 and 33.7 months, respectively (HR 0.78) [27].

Finally, ribociclib received approval, based on the MONALEESA-7 results, for the association with an aromatase inhibitor or tamoxifene in premenopausal patients. This is the first trial addressing young premenopausal metastatic breast cancer patients. In this study, 672 patients with up to one line of previous chemotherapy and no endocrine therapy in the metastatic setting were randomized to receive ribociclib or placebo plus endocrine therapy. Median PFS, the primary endpoint, had an improvement of 11 months in the experimental arm (23.8 vs. 13.0 months, respectively, HR 0.55, *p* < 0.0001). Serious adverse events were reported in 18% and 12% of patients, respectively [28]. An updated analysis with a median follow-up of 53.5 months reported a median OS with ribociclib plus endocrine treatment of 58.7 months vs. 48.0 months with the placebo arm, with a 24% reduction of risk of death with the experimental treatment (HR 0.76) [29].

The third CDK4/6 inhibitor approved was abemaciclib, indicated for the treatment of women with hormone receptor-positive, HER2-negative locally advanced or metastatic breast cancer in combination with an aromatase inhibitor or fulvestrant as an initial endocrine-based therapy, or in women who received prior endocrine therapy. The approvals were based on the MONARCH 2 and 3 trials, respectively. In the first study, abemaciclib in association with fulvestrant was tested in 669 hormone receptor-positive and HER2-negative breast cancer patients who progressed after endocrine therapy. The study showed a good safety profile associated with an improvement in PFS, resulting in 16.4 months for the abemaciclib arm vs. 9.3 months for fulvestrant plus placebo (HR 0.55, *p* < 0.001). The most common adverse events in the experimental arm were gastrointestinal adverse events (diarrhea: 86.4% vs. 24.7% and nausea: 45.1% vs. 22.9%), neutropenia (46.0% vs. 4.0%), and fatigue (39.9% vs. 26.9%) [22]. An update of the trial at 47.7 months of follow-up showed an improvement of 9 months in OS (46.7 months for abemaciclib plus fulvestrant and 37.3 months for placebo plus fulvestrant, HR 0.76, *p* = 0.01) [23].

In the first-line setting, abemaciclib was tested in association with a non-steroidal aromatase inhibitor (anastrozole or letrozole) in the MONARCH 3 trial. The study showed an improvement in the primary endpoint PFS (not reached in the experimental arm and 14.7 months in the placebo arm, HR 0.54, *p* = 0.000021). The most frequent grade 3 or 4 adverse events reported in the abemaciclib arm were neutropenia (21.1% vs. 1.2%) and leukopenia (7.6% vs. 0.6%) and drug-specific toxicity diarrhea (9.5% vs. 1.2%) [24]. The trial update at 26.7 months of follow-up confirmed a significantly longer median PFS with abemaciclib (28.2 versus 14.8 months; HR 0.54, *p* = 0.000002), with a longer duration of response (27.4 versus 17.5 months, respectively) [25].

The only endocrine therapy that received approval between the analyzed period was fulvestrant, which obtained two different extensions of indication: the first based on the PALOMA-3 trial results in association with palbociclib [11], while the second for the treatment of endocrine therapy-naive hormone receptor-positive breast cancer, based on FALCON trial results. In this study, 462 patients were randomized to receive fulvestrant or anastrozole. The primary endpoint was PFS, which was significantly longer in the fulvestrant group than in the anastrozole group (median PFS 16.6 months vs. 13.8 months, respectively, HR 0.80, *p* = 0.0486), with a similar toxicity profile. In particular, the most common adverse events were arthralgia and hot flushes (17% vs. 10% and 11% vs. 10% in the fulvestrant and anastrozole group, respectively), with a discontinuation rate of 7% in the fulvestrant group and 5% in the anastrozole group [14].

##### Hormone Receptor Positive/HER2 Negative with PIK3CA Mutations

The last approval concerned hormone receptor-positive and HER2-negative metastatic breast cancer with PIK3CA mutations, where alpelisib received indication in combination with fulvestrant for the treatment of postmenopausal women and men after disease progression following endocrine therapy as monotherapy (in the pivotal SOLAR-1 trial, only about 6% of patients previously received CDK4/6 inhibitors). In the randomized phase III trial, alpelisib or placebo were administered both in PIK3CA-mutated and non-mutated (proof-of-concept analysis) patients in association with fulvestrant. The PIK3CA mutations evaluated in the trial were 12C420R, E542K, E545A, E545D (1635G > T only), E545G, E545K, Q546E, Q546R, H1047L, H1047R, and H1047. In PIK3CA-mutated patients receiving alpelisib or placebo with fulvestrant, the median PFS (primary endpoint) was 11 and 5.7 months, respectively (HR 0.65, *p* < 0.001). In the cohort of non-mutated patients, proof-of-concept criteria were not met. Grade ≥ 3 adverse events were hyperglycemia (36.6%), rash (9.9%), and diarrhea (6.7%) [37]. The final OS results at 42.4 months of follow-up showed an advantage of 7.9 months (39.3 vs. 31.4 months, HR 0.86, *p* = 0.15) for the alpelisb arm but did not cross the prespecified efficacy boundary [38].

#### 3.2.2. HER2 Positive

In the HER2-positive setting, the three approvals regarded tucatinib, trastuzumab deruxtecan, and trastuzumab/pertuzumab subcutaneous formulation.

Both tucatinib and trastuzumab deruxtecan are indicated for the treatment of adult patients with HER2-positive locally advanced or metastatic breast cancer who have received at least two prior anti-HER2 treatment regimens. In particular, the oral tyrosine kinase inhibitor tucatinib has been approved in association with trastuzumab and capecitabine, based on the HER2CLIMB trial. In this study, patients progressed after trastuzumab, pertuzumab, and trastuzumab emtansine were randomized 2:1 to receive tucatinib or placebo in association with trastuzumab and capecitabine. The trial showed an improvement in the primary endpoint PFS (7.8 vs. 5.6 months, HR 0.54, *p* < 0.001) and in the secondary endpoint OS (21.9 vs. 17.4 months, HR 0.66, *p* = 0.005). Regarding safety, the tucatinib regimen caused an increase of grade ≥ 3 diarrhea and aminotransferase levels than those reported in the control group [40].

The antibody-drug conjugate trastuzumab deruxtecan was approved based on the phase II DESTINY-Breast01 trial. The study was composed of two parts: the first to establish the recommended dose, with a result of 5.4 mg per kilogram of body weight, and the second to determine the efficacy and safety of the chosen regimen. The primary endpoint was the objective response rate (ORR) of patients who were treated with the recommended dose, evaluated by an independent central review. In total, 184 patients pretreated with a median of six previous therapies (mandatory trastuzumab emtansine) received trastuzumab deruxtecan with 60.9% of ORR (6% complete response, CR), a median duration of response of 14.8 months, median duration PFS of 16.4 months, and OS not reached. The most common grade ≥ 3 adverse events were myelosuppression (neutropenia in 20.7% and anemia in 8.7%) and gastrointestinal events (nausea in 7.6%). Of note, 13.6% of patients reported an interstitial lung disease (10.9% grade 1–2, 0.5% grade 3–4, and 2.2% grade 5) [41].

Finally, in the setting of HER2-positive metastatic disease, the fixed-dose subcutaneous formulation of pertuzumab/trastuzumab was also approved, based on the FeDeriCa trial results [39]. Even though the study enrolled only patients in the early setting, the company also requested approval in metastatic disease, based on the current indications of pertuzumab.

#### 3.2.3. Triple Negative

##### Triple Negative with PD-L1 Positive

The only drug approved in this setting was the immune checkpoint inhibitor atezolizumab, evaluated in the IMpassion130 trial in association with nab-paclitaxel versus placebo and nab-paclitaxel in 902 triple-negative breast cancer patients. The two primary endpoints were PFS (in the intention-to-treat [ITT] population and PD-L1–positive subgroup) and OS (evaluated hierarchically in the ITT population and, if significant, in the PD-L1–positive subgroup). In the study, the experimental arm showed an advantage in PFS and a non-statistically significant improvement in OS in the ITT population (median PFS 7.2 vs. 5.5 months, HR 0.80, *p* = 0.002, median OS 21.3 vs. 17.6 months, HR 0.84, *p* = 0.08). Additionally, in the subgroup of patients with PD-L1 expression, which represented 40% of patients, an improvement of endpoints was shown (median PFS 7.5 vs. 5.0 months, HR 0.62, *p* < 0.001 and median OS 25.0 vs. 15.5 months, respectively, HR 0.62, even if a formal testing of OS in this group was not done for hierarchical statistical analysis, as previously mentioned). Regarding safety, grade ≥ 3 adverse events occurred in 48.7% vs. 42.2% of patients in the experimental and control group, respectively, and the most common were myelosuppression (neutropenia and anemia), fatigue, and neuropathy [34]. The update of the trial at 18.8 months of follow-up confirmed the non-statistically significant difference in OS in the ITT population (21.0 vs. 18.7 months, respectively, HR 0.87, *p* = 0.077) and the exploratory OS analysis in PD-L1-positive patients showed a difference of 7 months (25.4 vs. 17.9 months, *p* = 0.67) [35]. The EMA approval concerned only triple-negative breast cancer patients with PD-L1 ≥ 1%.

#### 3.2.4. BRCA 1/2 Mutation

In BRCA 1/2-mutated cancers, two PARP inhibitors obtained approval: olaparib and talazoparib. Both are approved in monotherapy for the treatment of BRCA-mutated HER2-negative locally advanced or metastatic breast cancer patients previously treated with an anthracycline and a taxane in the (neo)adjuvant or metastatic setting and endocrine therapy if hormone receptor positive.

Olaparib was tested in the OlympiAD trial, where 302 previously treated patients (no more than two previously chemotherapies and at least one hormonal therapy) with germline BRCA 1/2 mutation were randomized in a 2:1 ratio to receive the PARP inhibitor or the investigators’ choice chemotherapy with capecitabin, eribulin, or vinorelbine. Olaparib showed an advantage of 2.8 months in median PFS (HR 0.58, *p* < 0.001) with a good toxicity profile; in particular, grade 3 or higher adverse events were reported in 36.6% and 50.5% of patients in the experimental arm and standard chemotherapy, respectively, and the most common were hematologic events. The rate of treatment discontinuation for toxic effects was 4.9 and 7.7%, respectively. The OS had not yet reached 50% of the events, but it did not differ significantly (19.3 vs. 19.6 months, HR 0.90, *p* = 0.513) [30]. The update publication of the trial at a median follow-up of 25.3 and 26.3 months for the olaparib and the control arm, respectively, showed a non-statistically significant difference in OS of 2.2 months (HR 0.90, *p* = 0.513) [31].

Indeed, talazoparib was evaluated in the phase III EMBRACA trial in patients with germline BRCA 1/2 mutation, previously treated with no more than three lines of chemotherapies (no limits for previous hormonal therapy in the case of hormone receptor-positive breast cancer). In total, 431 patients were randomized in a 2:1 ratio to receive talazoparib or the physicians’ choice chemotherapy (capecitabine, eribulin, gemcitabine, or vinorelbine). Median PFS, which was the primary endpoint, was 8.6 versus 5.6 months, respectively (HR 0.54, *p* < 0.001), and OS, one of the secondary endpoints, was 22.3 and 19.5 months (HR 0.76, *p* = 0.11). The incidence of grade 3–4 hematologic adverse events was higher in the experimental arm (55% vs. 38%), while grade 3 non-hematologic adverse events occurred in 32% and 38% of patients, respectively [32]. The updated OS final results at a median follow-up of 44.9 and 36.8 months for the talazoparib and the chemotherapy arm, respectively, showed no difference in OS (19.3 vs. 19.5 months, HR 0.85, *p* = 0.17) [33].

## 4. Discussion

Breast cancer, along with lung cancer and melanoma, is one of the principal tumors for which new drugs have been approved by EMA in the last years.

Our analysis showed that only one approval in the locally advanced setting and one in the metastatic disease were based on phase 2 trials and none on phase 1 trials.

The majority of approvals were in metastatic disease (76%); in this setting, more than 60% were issued in hormone receptor-positive/HER2-negative patients and of them, 80% concerned a CDK4/6 inhibitor. Instead, in localized and locally advanced tumors, all approvals concerned HER2-positive disease.

**Figure 1:**
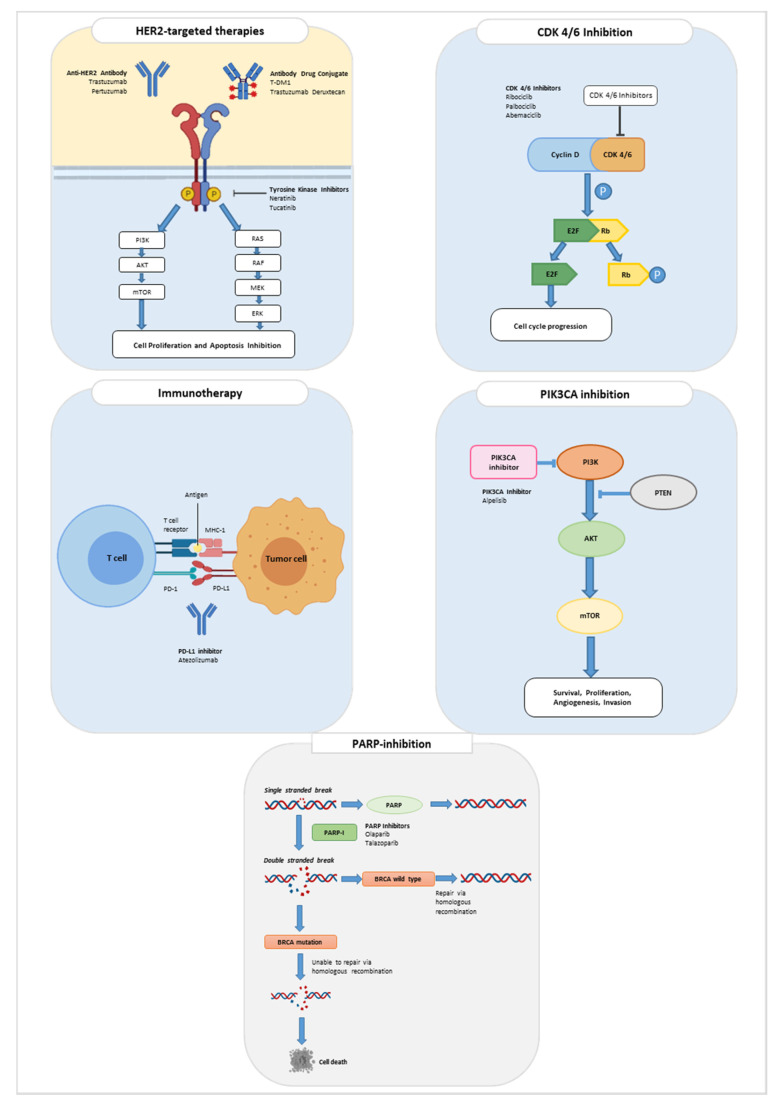
Therapeutic targets of new drugs approved for the treatment of breast cancer. In this figure, the mechanism of action of new approved drugs is shown. In particular, the new approved therapies belong to the category of HER2-targeted drugs, CDK 4/6 inhibitors, immunotherapy, PIK3CA inhibitors, and PARP inhibitors.

An interesting consideration is that, after more than 20 years of trastuzumab approval, new drugs have entered the breast cancer treatment landscape based on biomarker expression. In particular, for the first time in triple-negative disease, two biomarkers are considered in clinical practice: PD-L1 and BRCA. In luminal tumors, after over 50 years, new biological molecules have been approved other than endocrine therapy and a new biomarker, PIK3CA, is being evaluated for specific treatment. Finally, for HER2-positive tumors, HER2 expression remained the unquestioned target, with two new drugs, the tyrosine kinase inhibitor tucatinib and the antibody drug conjugate trastuzumab deruxtecan, that entered the therapeutic scenario of this disease (Figure 1).

Regarding primary endpoint, among 20 clinical trials leading to the 21 approvals, only one considered OS as a primary endpoint [34,35]. In particular, in trials concerning early disease, the primary endpoint was IDFS for the adjuvant setting and pCR [5,6], safety [7,8], and pharmacokinetics (PK) [39] for neoadjuvant trials. In advanced disease, the primary endpoint was PFS in all cases, except for the phase II Destiny Breast01 trial, in which the primary endpoint was ORR [41] and for the phase III IMpassion130 trial, in which PFS and OS were both primary endpoints [7,8].

Concerning clinical efficacy, almost all trials demonstrated an important improvement in the outcome. In the adjuvant setting, the experimental arm showed a reduction of 19% and 33% for 2-year IDFS in the APHINITY and ExteNET trials [18,19,20,21], respectively, and of 50% for 3-year IDFS in the KATHERINE study [36]. On the other hand, in the neoadjuvant setting, pertuzumab associated with trastuzumab improved pCR by 29.0% to 46.0% [5,6], and the new subcutaneous formulation of fixed-dose pertuzumab/trastuzumab showed a comparable pCR rate with respect to the intravenous regimen [39].

In metastatic disease, the CDK4/6 inhibitors associated with endocrine therapy radically changed clinical practice due to a remarkable improvement in terms of PFS compared to hormone therapy alone of 9–13 months in first line [9,10,15,16,24,25], of 8–11 months in both first second line [26,27,28,29]), and of 5–7 months in second line [11,12,13,22,23]. Those results have never been seen before and so this class of drugs entered quickly into clinical practice. In hormone receptor-positive tumors, fulvestrant also showed a benefit in PFS of almost 3 months versus anastrozole when used in first line [14]. In resistant aromatase inhibitors tumors, alpelisb showed an advantage in PFS of more than 5 months only in PIK3CA-mutated patients [37,38]. In HER2-positive pretreated patients, tucatinib improved PFS by about 2 months [40] and in heavily pretreated patients, trastuzumab deruxtecan showed an ORR of about 61% associated with a very interesting PFS of 16.4 months [41]. One of the most exciting evolutions in the last two years in the breast cancer therapeutic landscape regards the access of checkpoint inhibitors. Indeed, atezolizumab associated with nab-paclitaxel showed an increase of 2.5 months in terms of PFS in PD-L1-positive disease [34,35], so EMA approved the drug in this subset of patients. Finally, in BRCA-mutated patients, the PARP inhibitors allowed an improvement in PFS of about 3 months to be obtained [30,31,32,33].

Among 14 trials leading to drug approval in metastatic breast cancer, only three reported OS results at the approval time. In particular, tucatinib showed a significant advantage of 4.5 months [40], atezolizumab demonstrated a non-statistically improvement of 3.7 months in the ITT population and an interesting clinical advantage of 9.5 months in PD-L1-positive patients [34,35], and talazoparib did not demonstrate a statistically significant benefit in OS [32,33]. The trials with an available OS update were those that evaluated CDK4/6 inhibitors in both the first and second line and second line, showing an improvement of 11–12 and 7–9 months, respectively [11,12,13,22,23,26,27,28,29]. The other trials were SOLAR-1, in which alpelisib showed a non-statistically significant benefit in OS (8 months) [37,38], and the trials with PARP-inhibitors, where an advantage in OS was not demonstrated [32,33].

## 5. Conclusions

Considering the high incidence of breast cancer, medical and commercial interest focused on this disease has led to an investment in research in this pathology. A lot of innovative drugs have been approved for the treatment of breast cancer in the last years, with the majority of cases showing impressive results in terms of PFS and/or OS. In particular, the big novelty of locally advanced breast cancer treatment was the introduction of the HER2 double block, so that clinicians can now be confident in treating an earlier stage of cancer with a pre-operative strategy. In advanced luminal breast cancer, CDK4/6 inhibitors demonstrated a remarkable improvement in PFS and OS, and recently, the biomarkers PIK3CA and BRCA have defined a new strategy of treatment. In advanced HER2-positive tumors, we have new sequences due to the evidence of the significant impact of PFS with new molecules from the third line. In triple-negative breast cancer, the two new biomarkers, BRCA and PD-L1, have led to the use of specific therapeutic strategies.

Finally, some drugs have demonstrated little improvement in clinical outcomes, in some cases without an OS advantage. However, other factors, such as quality of life, toxicities, manageability, and costs (direct and indirect), should be considered. Moreover, a regular safety and efficacy monitoring of approved drugs should also be organized to establish the real benefit of the new indication.

In conclusion, thanks to these approvals, we are seeing a magnitude of clinical benefits for breast cancer patients. However, a careful selection of those who could really benefit from a treatment through the identification of biomarkers predictive of efficacy should be recommended.

## Figures and Tables

**Table 1:** New indications of breast cancer drugs issued by EMA between January 2015 and June 2021 and pivotal clinical trials.

Year of Approval	Drug	NM/EI	Trial	Setting	Biological Subgroup
2015	Pertuzumab	EI	NeoSphere [5,6], TRYPHAENA [7,8]	Localized/Locally Advanced	HER2+
2016	Palbociclib	NM	PALOMA-2 [9,10]	Metastatic	Luminal
2016	Palbociclib	NM	PALOMA-3 [11,12,13]	Metastatic	Luminal
2017	Fulvestrant	EI	FALCON [14]	Metastatic	Luminal
2017	Ribociclib	NM	MONALEESA-2 [15,16]	Metastatic	Luminal
2017	Fulvestrant	EI	PALOMA-3	Metastatic	Luminal
2018	Pertuzumab	EI	APHINITY [17,18]	Localized/Locally Advanced	HER2+
2018	Neratinib	NM	ExteNET [19,20,21]	Localized/Locally Advanced	HER2+
2018	Abemaciclib	NM	MONARCH 2 [22,23]	Metastatic	Luminal
2018	Abemaciclib	NM	MONARCH 3 [24,25]	Metastatic	Luminal
2018	Ribociclib	EI	MONALEESA-3 [26,27]	Metastatic	Luminal
2018	Ribociclib	EI	MONALEESA-7 [28,29]	Metastatic	Luminal
2019	Olaparib	EI	OlympiAD [30,31]	Metastatic	Luminal and triple negative with BRCAm
2019	Talazoparib	NM	EMBRACA [32,33]	Metastatic	Luminal and triple negative with BRCAm
2019	Atezolizumab	EI	IMpassion130 [34,35]	Metastatic	Triple negative
2019	Trastuzumab Emtansine	EI	KATHERINE [36]	Localized/Locally Advanced	HER2+
2020	Alpelisib	NM	SOLAR-1 [37,38]	Metastatic	Luminal
2020	Trastuzumab/pertuzumab sc	NM	FeDeriCa [39]	Localized/Locally Advanced	HER2+
2020	Trastuzumab/pertuzumab sc	NM	FeDeriCa [39]	Metastatic	HER2+
2020	Tucatinib	NM	HER2CLIMB [40]	Metastatic	HER2+
2020	Trastuzumab deruxtecan	NM	DESTINY-Breast01 [41]	Metastatic	HER2+

NM = new medicine, EI = extension of indication, HER2 = human epidermal growth factor receptor 2, BRCAm = BReast CAncer gene mutation.

**Table 2:** Results of clinical trials (first analysis and update) leading to approval for the treatment of localized/locally advanced breast cancer.

Trial	Phase	Setting	Target	Hormonal Receptor Status (ER and/or PgR+; %)	Experimental Arm	Control Arm	Primary Endpoint	Results	Hazard Ratio	*p*	Update
NeoSphere [5,6]	II	Neoadjuvant	HER2	47	ARM B H + P + T ARM C H + P ARM D P + T	ARM A H + T	pCR	ARM A 29.0% ARM B 45.8% ARM C 24.0% ARM D 16.8%	NA	NA	5-year PFS: ARM A 81% ARM B 86% ARM C 73% ARM D 73%
TRYPHAENA [7,8]	II	Neoadjuvant	HER2	51	ARM A FEC + H + P × 3→T + H + P × 3 ARM B FEC × 3→T + H + P × 3 ARM C TCH + P × 6	NA	Safety	Symptomatic LVSD: ARM A 0% ARM B 2.7% ARM C 0% Declines in EF ≥ 10% to <50%: ARM A 5.6% ARM B 5.3% ARM C 3.9%	NA	NA	3 years PFS: ARM A 89% ARM B 89% ARM C 87% Any-grade LVSD: ARM A 2.8% ARM B 4.0% ARM C 5.4% Declines in EF ≥ 10% to <50%: ARM A 11.1% ARM B 16.0% ARM C 11.8%
APHINITY [17,18]	III	Adjuvant	HER2	64	CHT + H + P	CHT + H+placebo	IDFS	3-year IDFS: 94.1% vs. 93.2%	0.81	*p* = 0.045	6-year IDFS: 91% vs. 88% 6-year OS: 95% vs. 94%
ExteNET [19,20,21]	III	Adjuvant	HER2	57 *	Neratinib	Placebo	IDFS	2-year IDFS: 93.9% vs. 91.6%	0.67	*p* = 0·0091	5-year IDFS: 90.2% vs. 87.7% Hormone receptor positives: IDFS: 90.8% vs. 85.7%, OS: 91.5% vs. 89.4%
KATHERINE [36]	III	Adjuvant	HER2	72	T-DM1	Trastuzumab	IDFS	3 years IDFS: 88.3% vs. 77.0%	0.50	*p* < 0.001	-
FeDeriCa [39]	III	Neoadjuvant	HER2	62	Fixed dose H/P sc	H + P iv	PK	Ratio P serum Ctrough sc to iv: 1.22	NA	NA	-

ER = estrogen receptor, PgR = progesterone receptor, HER2 = human epidermal growth factor receptor 2, H = trastuzumab, P = pertuzumab, T = docetaxel, pCR = pathologic complete response, NA = not applicable, PFS = progression free survival, FEC = 5-fluorouracil, epirubicin, cyclophosphamide, C = carboplatin, LVSD left ventricular systolic dysfunction, EF = ejection fraction, CHT = chemotherapy, IDFS = invasive disease-free survival, OS = overall survival, PK = pharmacokinetics. * Approval concerned the hormone receptor-positive subgroup.

**Table 3:** Results of clinical trials (first analysis and update) leading to the approval for the treatment of metastatic breast cancer.

Trial	Phase	Target	Biological Features (%)	Experimental Arm	Control Arm	Line	Primary Endpoint	PFS (mos)	Hazard Ratio	*p*	Delta	Update PFS (mos)	OS (mos)	Hazard Ratio	*p*	Delta	Update OS (mos)
PALOMA-2 [9,10]	III	HR+	ER: 100 PgR: NS	Palbociclib + letrozole	Placebo + letrozole	1	PFS	24.8 vs. 14.5	0.58	*p* < 0.001	10.3	27.6 vs. 14.5	NR vs. NR	NA	NA	NA	-
PALOMA-3 [11,12,13]	III	HR+	ER: 93 PgR: 66	Palbociclib + fulvestrant	Placebo + fulvestrant	2	PFS	9.2 vs. 3.8	0.42	*p* < 0.001	5.4	9.5 vs. 4.6	NR vs. NR	NA	NA	NA	34.9 vs. 28.0
MONALEESA-2 [15,16]	III	HR+	ER: 100 PgR: 82	Ribociclib + letrozole	Placebo + letrozole	1	PFS	NR vs. 14.7	0.56	*p* = 3.29 × 10^−6^	NA	25.3 vs. 16.0	NR vs. NR	NA	NA	NA	-
MONALEESA-3 [26,27]	III	HR+	ER: 99 PgR: 71	Ribociclib + fulvestrant	Placebo + fulvestrant	1–2	PFS	20.5 vs. 12.8	0.59	*p* < 0.001	7.7	-	NR vs. NR	NA	NA	NA	53.7 vs. 41.5
MONALEESA-7 [28,29]	III	HR+	ER: 99 PgR: 86	Ribociclib + NSAI or tamoxifene	Placebo + NSAI or tamoxifene	1–2	PFS	23.8 vs. 13.0	0.55	*p* < 0.0001	10.8	-	NR vs. NR	NA	NA	NA	58.7 vs. 48.0
MONARCH-2 [22,23]	III	HR+	ER: NS PgR: 76	Abemaciclib + fulvestrant	Placebo + fulvestrant	2	PFS	16.4 vs. 9.3	0.55	*p* < 0.001	7.1	-	NR vs. NR	NA	NA	NA	46.7 vs. 37.3
MONARCH-3 [24,25]	III	HR+	ER: NS PgR: 77	Abemaciclib + NSAI	Placebo + NSAI	1	PFS	NR vs. 14.7	0.54	*p* = 0.000021	NA	28.2 vs. 14.8	NR vs. NR	NA	NA	NA	-
FALCON [14]	III	HR+	ER: 99 PgR: 76	Fulvestrant	Anstrozole	1	PFS	16.6 vs. 13.8	0.80	*p* = 0.0486	2.8	-	NR vs. NR	NA	NA	NA	-
SOLAR-1 [37,38]	III	HR+	ER and PgR: NS PIK3CAm: 60 *	Alpelisib	Placebo	2	PFS	11.0 vs. 5.7	0.65	*p* < 0.001	5.3	-	NR vs. NR	NA	NA	NA	39.3 vs. 31.4
HER2CLIMB [40]	III	HER2+	ER and/or PgR: 62	Tucatinib + trastuzumab + capecitabine	Placebo + trastuzumab+ capecitabine	≥3	PFS	7.8 vs. 5.6	0.54	*p* < 0.001	2.2	-	21.9 vs. 17.4	0.66	*p* = 0.005	4.5	-
DESTINY-Breast01 [41]	II	HER2+	ER and/or PgR: 53	Trastuzumab deruxtecan	NA	≥3	ORR	16.4 vs. NA	NA	NA	NA	-	NR	NA	NA	NA	-
IMpassion130 [34,35]	III	TN	PD-L1 ≥ 1%: 40 **	Atezolizumab + Nab-paclitaxel	Placebo + Nab-paclitaxel	1	PFS and OS	7.2 vs. 5.5 PD-L1+: 7.5 vs. 5.0	0.80 PD-L1+: 0.62	*p* = 0.002 PD-L1+: *p* < 0.001	1.7 PD-L1+: 2.5	-	21.3 vs. 17.6 PD-L1+: 25.0 vs. 15.5	0.84 PD-L1+: 0.62	*p* = 0.08	3.7 PD-L1+: 9.5	21.0 vs. 18.7 PD-L1+: 25.4 vs. 17.9
OlympiAD [30,31]	III	BRCAm	ER and/or PgR: 50 TN: 50 BRCA1m: 55 BRCA2m: 45	Olaparib	Investigators’ choice CHT	≥1	PFS	7.0 vs. 4.2	0.58	*p* < 0.001	2.8	-	NR vs. NR	0.90	*p* = 0.513	NA	19.3 vs. 17.1
EMBRACA [32,33]	III	BRCAm	ER and/or PgR: 56 TN: 44 BRCA1m: 45 BRCA2m: 55	Talazoparib	Investigators’ choice CHT	≥1	PFS	8.6 vs. 5.6	0.54	*p* < 0.001	3	-	22.3 vs. 19.5	0.76	*p* = 0.11	NA	19.3 vs. 19.5

PFS = progression free survival, OS = overall survival, HR = hormone receptor, ER = estrogen receptor, PgR = progesterone receptor, NS = not shown, NR = not reached, NA = not applicable, NSAI = non-steroidal aromatase inhibitors, PIK3Cam = phosphatidylinositol-4,5-bisphosphate 3-kinase, catalytic subunit alpha mutation, TN = triple negative, PD-L1 = programmed death-ligand 1, BRCAm = BReast CAncer gene mutation, CHT = chemotherapy. * Approval concerned only the PIK3CA-mutated subgroup. ** Approval concerned only the PD-L1-positive subgroup.
